# Access and time to chemotherapy among Ethiopian cancer patients: a population-based registry study

**DOI:** 10.1093/oncolo/oyag198

**Published:** 2026-05-20

**Authors:** Eva S M Hermann, Edom Seife, Pablo S C Santos, Eric S Kroeber, Solomon Asmare, Sophie Roßner, Andreas Wienke, Marcus Heise, Abdu A Yesufe, Adamu Addissie, Mathewos Assefa, Eva J Kantelhardt

**Affiliations:** Global and Planetary Health Working Group, Institute for Medical Epidemiology, Biometrics and Informatics, Martin Luther University Halle-Wittenberg, Halle (Saale) 06112, Germany; Department of Oncology, Tikur Anbessa Specialized Hospital, Addis Ababa University, Addis Ababa 5657, Ethiopia; Global and Planetary Health Working Group, Institute for Medical Epidemiology, Biometrics and Informatics, Martin Luther University Halle-Wittenberg, Halle (Saale) 06112, Germany; Global and Planetary Health Working Group, Institute for Medical Epidemiology, Biometrics and Informatics, Martin Luther University Halle-Wittenberg, Halle (Saale) 06112, Germany; Addis Ababa City Cancer Registry, Addis Ababa, Ethiopia; Global and Planetary Health Working Group, Institute for Medical Epidemiology, Biometrics and Informatics, Martin Luther University Halle-Wittenberg, Halle (Saale) 06112, Germany; Global and Planetary Health Working Group, Institute for Medical Epidemiology, Biometrics and Informatics, Martin Luther University Halle-Wittenberg, Halle (Saale) 06112, Germany; Global and Planetary Health Working Group, Institute for Medical Epidemiology, Biometrics and Informatics, Martin Luther University Halle-Wittenberg, Halle (Saale) 06112, Germany; Institute of General Practice and Family Medicine, Medical Faculty, Martin-Luther-University Halle-Wittenberg, Halle (Saale) 06112, Germany; Department of Oncology, St. Paul Hospital Millennium Medical College, Addis Ababa 1271, Ethiopia; Global and Planetary Health Working Group, Institute for Medical Epidemiology, Biometrics and Informatics, Martin Luther University Halle-Wittenberg, Halle (Saale) 06112, Germany; School of Public Health, Addis Ababa University, Addis Ababa 9086, Ethiopia; Global and Planetary Health Working Group, Institute for Medical Epidemiology, Biometrics and Informatics, Martin Luther University Halle-Wittenberg, Halle (Saale) 06112, Germany; Department of Oncology, Tikur Anbessa Specialized Hospital, Addis Ababa University, Addis Ababa 5657, Ethiopia; Addis Ababa City Cancer Registry, Addis Ababa, Ethiopia; Global and Planetary Health Working Group, Institute for Medical Epidemiology, Biometrics and Informatics, Martin Luther University Halle-Wittenberg, Halle (Saale) 06112, Germany; Department of Gynecology, Martin Luther University Halle-Wittenberg, Halle (Saale), Germany

**Keywords:** Ethiopia, sub-Saharan Africa, access to care, cancer, treatment barriers, chemotherapy, oncology services

## Abstract

**Background:**

Chemotherapy is an essential component of comprehensive cancer care and timely access can improve survival outcomes. The objective of this study was to assess the time to chemotherapy initiation and patient-reported barriers to cancer care among patients from Addis Ababa, Ethiopia.

**Methods:**

We conducted a retrospective study using a randomly selected sample of 350 patients with common cancers (breast [ICD-10: C50], cervical [C53], colorectal [C18-20], esophageal [C15], gastric [C16], and ovarian [C56]) registered at the population-based Addis Ababa City Cancer Registry in 2021. Patients or their relatives were followed-up via questionnaire-based telephone interview and treatment data were extracted from their medical records. The time from pathological diagnosis to initiation of chemotherapy (time to treatment) was calculated. Regression analyses were conducted to identify predictors of chemotherapy initiation and treatment delays.

**Results:**

Most patients (71.7%) received chemotherapy and the median time to treatment was 63 days (IQR 36.5-128 days). Patients reported considerable barriers to treatment regarding fear, cost and waiting times. Having health insurance (OR 2.70; 95% CI, 1.33-5.50) and trust in healthcare professionals (OR 1.76; 95% CI, 1.16-2.68) were associated with higher odds of receiving chemotherapy. Patients with cervical (OR 0.07; 95% CI, 0.03-0.18), gastric (OR 0.21; 95% CI, 0.05-0.86) or esophageal cancer (OR 0.10; 95% CI, 0.02-0.53) were less likely to initiate chemotherapy compared to breast cancer patients. Longer time to treatment was not associated with any of the assessed characteristics or perceived barriers.

**Conclusion:**

Over half of the cancer patients from Addis Ababa wait more than 2 months to initiate chemotherapy after a pathological diagnosis. Expanding oncology services and health insurance coverage could enhance access to treatment. Health professionals should promote patient trust by appropriately addressing their psychosocial needs, including cancer-related fear, to improve the uptake of treatment and thus clinical outcomes.

Implications for Practice Improving access to timely chemotherapy requires coordinated action across multiple levels. Strengthening health system capacity and care coordination is essential to reduce delays, while improving provider–patient communication and integrating psychosocial support can address fear and enhance treatment uptake and adherence. Expanding health insurance coverage may reduce financial barriers but must be accompanied by increased service availability to be effective.

## Background

In Sub-Saharan Africa (SSA), cancer incidence and mortality will likely double by 2040^1^ due to changing demographics and the spread of lifestyle related risk factors, while a high prevalence of infection-related cancers remains.^2^ This poses a considerable challenge to public health. Late-stage presentation^3^ and delays in diagnosis contribute to poorer survival rates in SSA compared to high-income countries.^4^ These disparities are further reinforced by inequalities in access to multimodal therapy: less than 15% of low-income countries have access to comprehensive cancer treatment, compared to 90% of high-income countries.^5^

Enhancing access to timely cancer treatment can improve cancer survival,^6^ as treatment delays of even 4 weeks are linked to adverse outcomes, the effect particularly clear in breast, colorectal and ovarian cancers.^7^^,^^8^ The World Health Organization recommends treatment initiation within 90 days after symptom recognition for all cancers,^9^ while acknowledging that reaching this threshold is challenging for many low- and middle-income countries.^10^

Chemotherapy is a crucial pillar of comprehensive cancer treatment, with special importance in low-resource settings, where advanced radiation technologies and targeted therapies are rare.^2^ With rising cancer numbers, the demand for chemotherapeutic drugs is growing.^11^ Still, access to essential chemotherapy drugs remains limited in many countries in SSA.^12^ The inconsistent availability of chemotherapy drugs is one of various challenges of healthcare systems struggling to cope with the rising patient volume. System-level barriers, like the lack of essential services (eg, pathology, diagnostic imaging, treatment), poorly trained staff, poor referral systems and inadequate infrastructure, bar patients from receiving timely cancer treatment.^13^^,^^14^

Few studies have been conducted in Sub-Saharan African countries, like Ethiopia, to assess treatment barriers faced by cancer patients and the reasons behind delays of cancer treatment, like chemotherapy. The purpose of this study was to assess the access to cancer care among patients registered in the Addis Ababa City Cancer Registry (AACCR), currently the only population-based registry in Ethiopia, to provide evidence for targeted interventions aiming to enhance treatment access. The objective was to evaluate the time from diagnosis to chemotherapy initiation and the patient-perceived barriers to cancer treatment to identify characteristics related to chemotherapy receipt and time to chemotherapy.

## Methods

### Setting and participants

We conducted a retrospective cohort study at the AACCR with 6 contributing health facilities: Tikur Anbessa Specialized Hospital (TASH), St. Paul’s Hospital Millennium Medical College (SPHMMC), United Vision Internal Medicine Specialty Center, St. Gabriel General Hospital, Girum General Hospital and the Lancet General Hospital.

The sample size was determined based on initial power calculation using the statsmodels v0.14.4 package for Python (functions *NormalIndPower* and *proportion_effectsize*),^15^ assuming that 50% of patients would experience delayed or no treatment, based on own previous research.^16^ The calculation yielded a minimum sample size of 500 patients (significance at 5% and power at 80%).

A random sample of 597 adult patients diagnosed in 2021 was selected from the AACCR database, allowing for approx. 20% loss to follow-up and missing records. Eligible patients had one of 6 common solid tumors with chemotherapy indications per National Comprehensive Cancer Networks’ (NCCN) Harmonized Guidelines^17^: breast (ICD-10: C50), cervical (C53), colorectal (C18-20), esophageal (C15), gastric (C16), and ovarian (C56) cancers. The NCCN publishes cancer treatment guidelines for resource-limited healthcare systems. After excluding patients diagnosed before 2021, with recurrent disease or prior cancers, 350 patients were included in downstream analyses. Medical records were obtained from 6 facilities, and patients or their relatives were followed up via telephone interviews between March and July 2023.

The specific impact of the COVID-19 pandemic on time to chemotherapy was not assessed, since Ethiopia’s low case numbers^18^ and absence of strict lockdowns have likely not affected access to cancer care. In addition, preliminary data from AACCR show no decline in cancer cases between 2020 and 2022.

### Objectives

The primary objective of this study was to assess the time to chemotherapy (treatment interval), defined as the time from the date of pathologically confirmed cancer diagnosis to the date of chemotherapy initiation. We aimed at understanding the patient-perceived barriers to treatment, by identifying factors associated with the probability of receiving chemotherapy and the length of the treatment interval.

### Data sources and measurement

Demographic data (gender, age), tumor characteristics (topography, stage), and diagnosis date were obtained from the AACCR’s database, which follows the Procedure Manual of the African Cancer Registry Network^19^ and defines diagnosis by first histological or cytological confirmation.

Medical records from 6 health facilities provided additional tumor and treatment data, including surgery and chemotherapy dates. Registry data were cross-checked and updated where necessary. The International Union for Cancer Control guidelines^20^ were used for stage classification and essential TNM guidelines applied when full staging was incomplete.^19^

### Questionnaire

At follow-up, patients or next-of-kin, participated in a structured telephone interview in Amharic, after giving oral consent. The questionnaire was adapted from a previous study in Addis Ababa^21^ and refined based on patient input after testing on randomly selected cancer patients. It included sociodemographic factors, treatment information and barriers to treatment.

Barriers were assessed using the 5 dimensions of access to care as defined by Penchansky et al.^22^: availability, accessibility, accommodation, affordability, and acceptability. Each dimension contained 2 questions, with responses rated on a 3-level Likert scale (*Very problematic*; *Somewhat problematic*; *Not problematic*) (Table S1).^13^^,^^14^^,^^22^ All participants answered all questions regardless of treatment status.

When medical records were incomplete, patients reported treatment dates using the Ethiopian calendar, later converted to the Gregorian calendar. For incomplete dates, standardized assumptions were applied (eg, mid-month defined as 15th or beginning, middle or end of year set as first of the third, seventh, or eleventh month), consistent across interview and record data.

### Statistics

Statistical analyses were performed using SPSS (v.28.0) and Python (statsmodels v0.14.4).^15^ Treatment intervals are reported as median with IQR and cumulative probabilities of initiating chemotherapy within 12 months were estimated and plotted. A logistic regression was used to assess the likelihood of receiving chemotherapy (results expressed as odds ratios [OR]). A negative binomial regression model was applied to evaluate factors influencing time to chemotherapy among treated patients, with results reported as incidence rate ratios (IRR). All estimates are presented with 95% CI and visualized using forest plots.

As a sensitivity analysis, Cox’s and Fine-Gray’s regression^23^ were applied to analyze the hazard of chemotherapy initiation, accounting for the scenario of death as a competing risk,^24^^,^^25^ with results expressed as sub-hazard ratio. Subgroup analysis was performed for breast cancer patients.

All models included sociodemographic, disease-specific factors and self-perceived barriers as explanatory variables. The negative binomial model (time to chemotherapy) was additionally adjusted for prior surgery.

To minimize data loss, a systematic imputation approach was used to infer missing barriers-related answers (1.3%) for patients with less than 3 missing answers. Following common practice,^26–28^ imputation was performed using predictors with strong association (*r*^2^ ≥ 0.6).

## Results

### Study participants

In this study, 350 patients were successfully traced, provided consent, and were therefore included (Figure S1). The majority of cases were reported by the 2 largest hospitals in Addis Ababa: 66.6% from Tikur Anbessa Hospital and 20.3% from St. Paul’s Hospital. Patients or their proxy were contacted after a median follow-up time of 22 months (IQR 19-25). At this time, two-thirds (*n *= 231; 66.0%) of patients were still alive. In 53.7% (*n *= 188) of cases, a next-of-kin answered the questionnaire—either as the patient had passed away (*n* = 119) or was unavailable to participate in the interview (*n* = 69).

The median age of the patients was 48 years (IQR = 37-59) at time of diagnosis, with a large female majority (*n *= 289; 82.6%) and breast cancer being the most prevalent (*n *= 148; 42.3%; Table 1). Nearly one-third (*n *= 107; 30.6%) lacked information on stage and many presented at advanced stages III (*n *= 99; 28.3%) and IV (*n *= 84; 24.0%; Table 2).

**Table 1:** Sociodemographic characteristics of the study cohort, given as median or *N* (column%).

Characteristics	Total cohort
	*N* = 350
**Age (median in years and grouped)**	48.0
** <= 35**	71 (20.3)
** 36-45**	92 (26.3)
** 46-55**	73 (20.9)
** 56-65**	74 (21.1)
** =>76**	10 (2.9)
**Sex**	
** Female**	289 (82.6)
** Male**	61 (17.4)
**Religion**	
** Orthodox Christian**	256 (73.1)
** Protestant**	35 (10.0)
** Muslim**	56 (16.0)
** Other**	3 (0.9)
**Marital status**	
** Married**	252 (72.0)
** Single**	39 (11.1)
** Divorced**	19 (5.4)
** Widowed**	40 (11.4)
**Educational level**	
** No schooling**	60 (17.1)
** Elementary**	118 (33.7)
** Secondary**	111 (31.7)
** Tertiary**	61 (17.4)
**Self-perceived wealth**	
** Poor**	139 (39.8)
** Middle class**	202 (57.7)
** Rich**	9 (2.6)
**Source of medical expenses^a^**	
** Out of pocket**	170 (48.6)
** Relatives**	314 (89.7)
** Health Insurance**	186 (53.1)
** Other form of payment**	31 (8.9)

a Percentages exceed 100% because of multiple answer options.

**Table 2:** Treatment characteristics, stage and survival status of the study cohort by cancer entity, given as median or *N* (Column%).

Treatment characteristics	Total cohort	Breast	Cervix uteri	Colorectum	Gastric	Ovary	Oesophagus
	*N* = 350	*N* = 148	*N* = 68	*N* = 64	*N* = 29	*N* = 21	*N* = 20
**Stage at diagnosis**							
** Stage I**	9 (2.6)	4 (2.7)	0 (0.0)	3 (4.7)	0 (0.0)	2 (9.5)	0 (0.0)
** Stage II**	51 (14.6)	26 (17.6)	14 (20.6)	8 (12.5)	1 (3.5)	1 (4.8)	1 (5.0)
** Stage III**	99 (28.3)	54 (36.5)	19 (27.9)	20 (31.3)	3 (10.3)	2 (9.5)	1 (5.0)
** Stage IV**	84 (24.0)	14 (9.5)	18 (26.5)	19 (29.7)	18 (62.1)	5 (23.8)	10 (50.0)
** Unknown**	107 (30.6)	50 (33.8)	17 (25.0)	14 (21.9)	7 (24.1)	11 (52.4)	8 (40.0)
**Treatment(s) received^a^**							
** Surgery**	223 (63.7)	128 (86.5)	11 (16.2)	48 (75.0)	12 (41.4)	17 (81.0)	7 (35.0)
** Chemotherapy**	251 (71.7)	128 (86.5)	29 (42.7)	48 (75.0)	18 (62.1)	17 (81.0)	11 (55.0)
** Radiotherapy**	78 (22.3)	35 (23.7)	33 (48.5)	9 (14.1)	0 (0.0)	0 (0.0)	1 (5.0)
** Endocrine treatment^b^**	86 (24.6)	86 (58.2)	—	—	—	—	—
Treatment combinations							
** No treatment**	51 (14.6)	9 (6.1)	20 (29.4)	5 (7.8)	8 (27.6)	3 (14.3)	6 (30.0)
** Surgery only**	29 (8.3)	9 (6.1)	5 (7.4)	8 (12.5)	3 (10.3)	1 (4.8)	3 (15.0)
** Chemotherapy only**	40 (11.4)	6 (4.1)	8 (11.8)	10 (15.6)	9 (31.0)	1 (4.8)	6 (30.0)
** Radiotherapy only**	11 (3.1)	1 (0.7)	10 (14.7)	0 (0.0)	0 (0.0)	0 (0.0)	0 (0.0)
** Surgery, chemotherapy, and radiotherapy**	34 (9.7)	29 (19.6)	0 (0.0)	5 (7.8)	0 (0.0)	0 (0.0)	0 (0.0)
** Surgery and chemotherapy**	152 (43.4)	89 (60.1)	2 (2.9)	32 (50.0)	9 (31.0)	16 (76.2)	4 (20.0)
** Surgery and radiotherapy**	8 (2.3)	1 (0.7)	4 (5.9)	3 (4.7)	0 (0.0)	0 (0.0)	0 (0.0)
** Chemotherapy and radiotherapy**	25 (7.1)	4 (2.7)	19 (27.9)	1 (1.6)	0 (0.0)	0 (0.0)	1 (5.0)
**Chemotherapy completion**							
** Yes**	206 (82.1)	114 (89.1)	24 (82.8)	34 (70.8)	13 (72.2)	14 (77.8)	7 (63.6)
** No**	36 (14.3)	13 (10.1)	2 (6.9)	13 (27.1)	3 (16.7)	3 (16.7)	2 (18.2)
** Unknown**	9 (3.6)	1 (0.8)	3 (10.3)	1 (2.1)	2 (11.1)	0 (0.0)	2 (18.2)
**Time from diagnosis to chemotherapy (median [IQR], in days)**	63 (36.5-128)	62 (39-106)	44 (16-95)	84 (47-142)	63 (30-201)	58 (14-192)	46 (29.5-127.5)
**Survival status at time of interview**							
** Alive**	231 (66.0)	126 (85.1)	39 (57.4)	35 (54.7)	9 (31.0)	16 (76.2)	6 (30.0)
** Deceased**	119 (34.0)	22 (14.9)	29 (42.6)	29 (45.3)	20 (69.0)	5 (23.8)	14 (70.0)

a Percentage referring to all patients per column.

b Breast cancer only.

Regarding economic status, 39.8% (*n *= 139) of patients regarded themselves as poor. Nearly 9 out of 10 (*n *= 314; 89.7%) reported relying on family and friends to cover the medical expenses and nearly half paying out of pocket (*n *= 170; 48.6%). Only about half had health insurance (*n *= 186; 53.1%).

### Treatment patterns and time to chemotherapy

Only 51 patients (14.6%) did not receive any form of cancer-directed therapy, with greatest share being women with cervical cancer (*n* = 20). In total, 251 (71.7%) initiated chemotherapy (Table 2), with a completion rate of 82.1% (*n *= 206). We found that more than half of breast cancer patients (86; 58.1%) received endocrine treatment.

**Figure 1.**
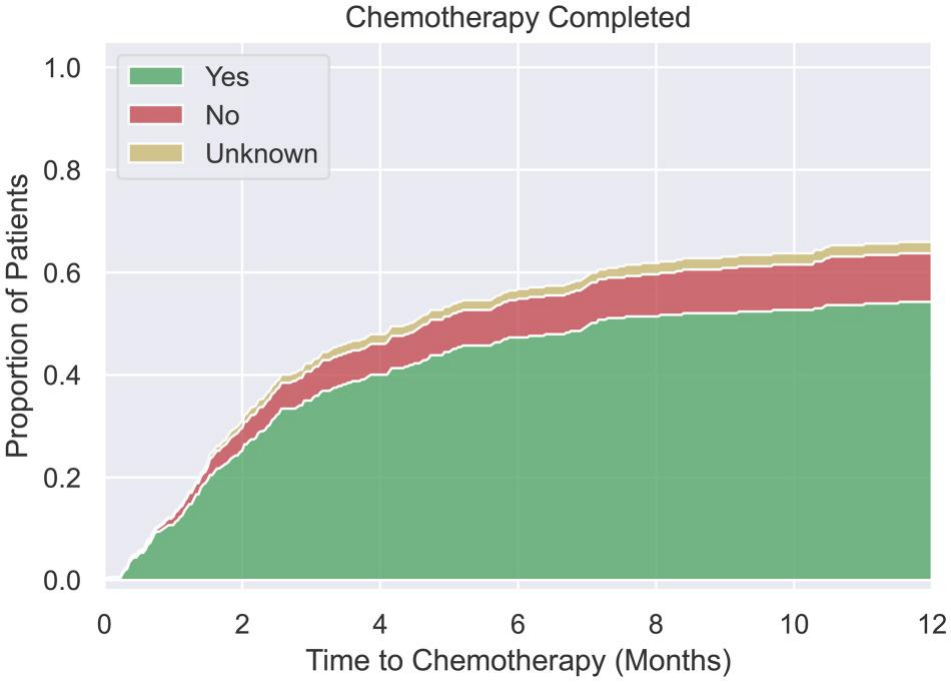
Proportion of patients receiving chemotherapy within 12 months, according to status of treatment completion. Unadjusted for death, patients with missing or implausible information on dates of chemotherapy were excluded.

Of all patients, 209 were eligible for time to treatment analysis. The median time to chemotherapy was 63 days (IQR 36.5-128 days). Only 19.1% of patients began chemotherapy within a month of their diagnosis, rising to 64.1% after 3 months and 65.9% after one year (Figure 1). Prior to chemotherapy, 140 (67.0%) patients underwent surgery, with a median time from surgery to chemotherapy of 68.5 days (IQR 48-100 days).

### Self-perceived barriers to treatment

**Figure 2.**
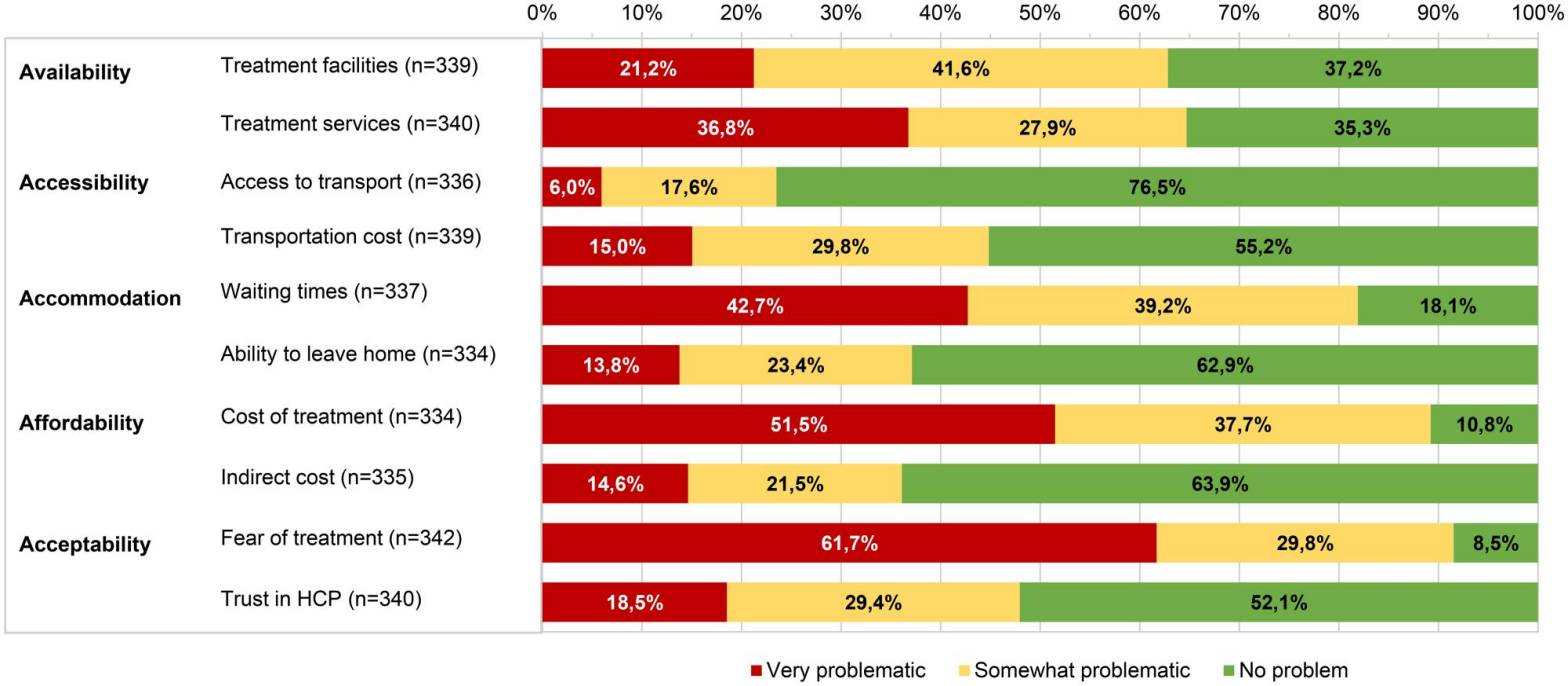
Self-reported barriers in access to cancer care and treatment across 5 dimensions of access to care (Penchansky et al.). For detailed description of each dimension and item, see Table S1.

The barrier reported as most problematic (Figure 2) was *fear of treatment* (*n* = 211; 61.7%; Figure 2). *Cost of treatment* (*n *= 172; 51.5%) and *waiting times* (*n *= 146; 42.4%) were regarded as very problematic by nearly half of patients.

Rated as least problematic was the *access to transportation* (*n *= 262; 76.2%). *Indirect cost of treatment* and *ability to leave home* were rated as not problematic by 64.2% (*n *= 221) and 63.7% (*n *= 219) of patients, respectively (Figure 2). Regarding *availability of treatment facilities and services*, participants gave mixed answers but the majority reported having at least some problems. Only every other participant (*n *= 177; 52.1%) reported no problems regarding *trust in healthcare professionals*.

### Factors associated with chemotherapy receipt

**Figure 3.**
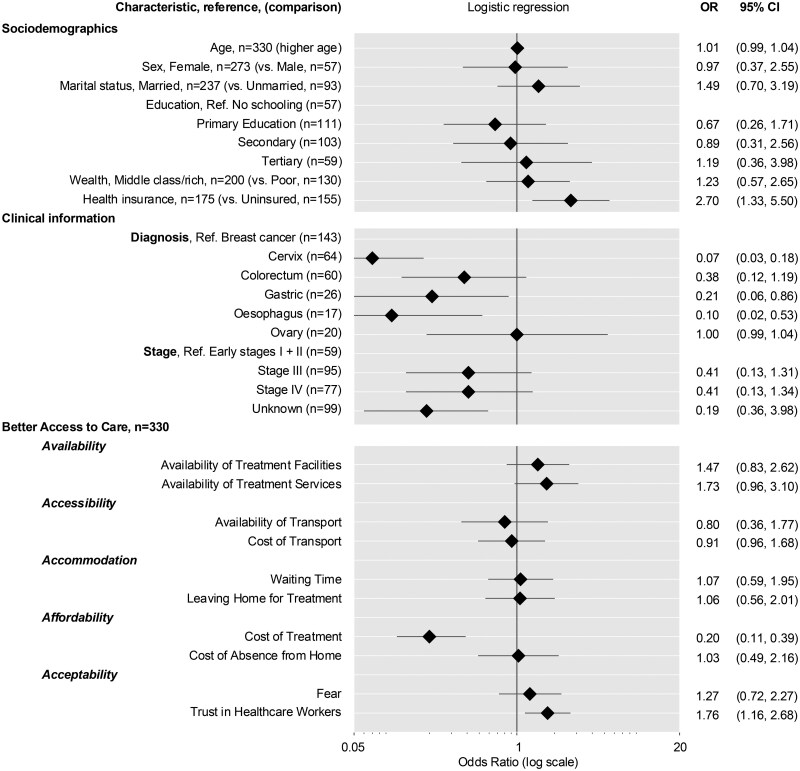
Forest plot of the odds of receiving chemotherapy (logistic regression). OR, odds ratio. OR > 1 indicates association with increased odds of receiving chemotherapy.

Cervical (OR 0.07; 95% CI, 0.03-0.18), gastric (OR 0.21; 95% CI, 0.05-0.86) or esophageal (OR 0.10; 95% CI, 0.02-0.53) cancer was associated with a decreased likelihood of receiving chemotherapy compared to patients with breast cancer (Figure 3; Table S2), so was missing stage information (OR 0.19; 95% CI, 0.06-0.59).

Having health insurance nearly tripled the odds of initiating chemotherapy (OR 2.7; 95% CI, 1.33-5.50). Reporting higher trust in healthcare providers increased chances of chemotherapy by 76% (OR 1.76; 95% CI, 1.16-2.68). Patients expressing fewer problems with availability of treatment services were more likely to receive chemotherapy (OR 1.73; 95% CI, 0.96-3.10). Rating the cost of treatment as less problematic decreased the likelihood of receiving the treatment (OR 0.20; 95% CI, 0.11-0.39).

The Fine-Gray regression results were consistent with the logistic regression, suggesting minimal bias from the competing risk of death (Table S3). Subgroup analysis in breast cancer patients showed similar findings (Table S4).

### Factors associated with time to chemotherapy initiation

**Figure 4.**
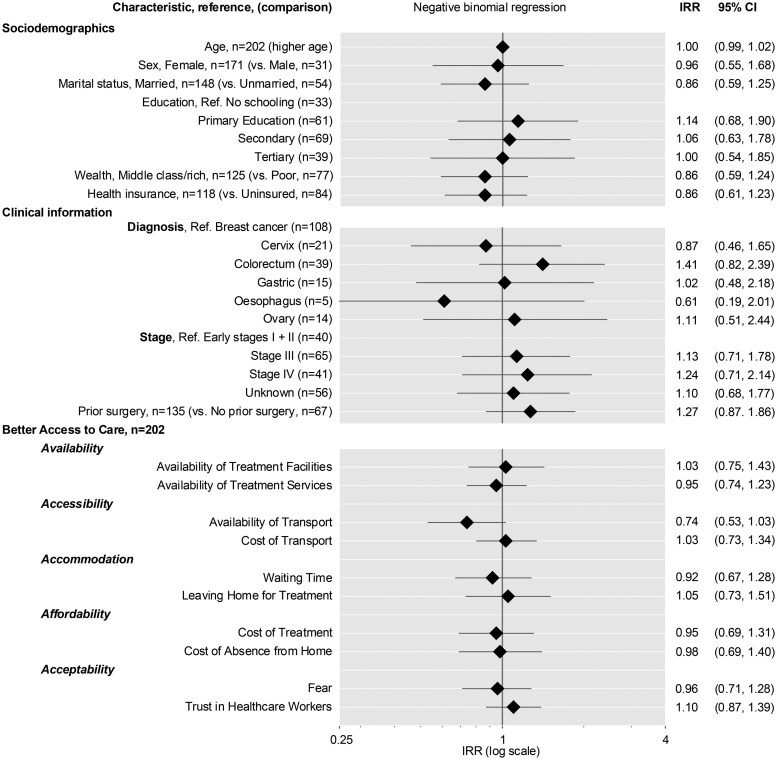
Forest plot of predictors of time interval to chemotherapy (negative binomial regression). IRR, incidence rate ratio. IRR > 1 indicates association with increased time to chemotherapy initiation.

Rating the access to transportation as less problematic was associated with shorter time to chemotherapy initiation (IRR 0.74; 95% CI, 0.53-1.03). Other than that, this model did not find any strong associations (Figure 4; Table S3).

In the subgroup analysis among breast cancer patients however, stage IV disease (IRR 2.60; 95% CI, 0.96-7.01) and prior surgery (IRR 1.75; 95% CI, 0.97-3.13) were associated with longer intervals. Being unmarried versus married (IRR 0.52; 95% CI, 0.28-0.97) and having primary-level education vs no schooling (IRR 0.46; 95% CI, 0.20-1.05) reduced the treatment interval by about half (Table S4).

### Effect of interviewee

As 54% of interviews were conducted with next-of-kin, we assessed potential bias from the responding person. To avoid overparameterization, responding person was not included in the main regression models. Instead, a separate logistic regression examined its association with reported barriers, followed by a sensitivity analysis. Logistic regression revealed no significant associations between assessed predictors and responding person, with all ORs close to 1 and 95% CI (Table S5). Sensitivity analysis indicated high power (99%) to detect large effects (OR = 2.0) and moderate power (70%) for medium effects (OR = 1.5), but low power (19%) for small effects (OR = 1.2). As no moderate or large effects were observed, these findings suggest no meaningful association between responding person and reported barriers in this dataset.

## Discussion

In a population-based approach, we assessed time to chemotherapy and patient-reported barriers to treatment among patients diagnosed with 6 common cancers in Addis Ababa. In our cohort, 71.7% of patients had received chemotherapy with an 82.1% completion rate. The median time to chemotherapy was 63 days and 64.1% of patients initiated chemotherapy within 3 months. The most commonly reported barriers to cancer treatment were fear of treatment, cost, availability of treatment services, and waiting times. Health insurance coverage and trust in health care professionals were facilitators of chemotherapy initiation.

### Treatment patterns and time to chemotherapy initiation

Two-thirds of patients in our cohort began chemotherapy, presenting progress compared a population-based study in 12 Sub-Saharan countries in 2012, where only around 55% of breast cancer^29^ and colorectal cancer^30^ patients received chemotherapy.

In our study, cervical, gastric and esophageal cancer patients were less likely to initiate treatment. This disparity may be attributed to the high prevalence of late-stage or unstaged disease or in these cancers (Table 1), limiting treatment options.^31^ Additionally, cervical cancer patients are often treated with concurrent radiochemotherapy.^17^ Due to a shortage of radiotherapy units and frequent machine breakdowns in Addis Ababa, waiting times for radiation are exceedingly long,^32^ potentially delaying or preventing concurrent chemotherapy. Addressing these structural issues requires investments in expanding radiotherapy infrastructure and optimizing patient referral pathways.

We found a median time to chemotherapy of 63 days, which is comparable to findings from South Africa^33^ and Rwanda.^34^ However, this timeframe is notably longer than in women with breast cancer from high-income settings such as the United States^35^^,^^36^ and Japan.^37^ In Addis Ababa, Gebremariam et al. reported that breast cancer patients waited a median of 63 days from surgery to adjuvant chemotherapy.^38^ The discrepancy between the time from diagnosis to chemotherapy (63 days) and the time from surgery to chemotherapy (68.5 days) in our study is likely due to the diagnostic workup and cancer registration process, as diagnosis is typically recorded at the time of pathology reporting rather than at biopsy or surgery.

Over the past decade, delays remained constant in Addis Ababa with increasing patient volume^18^: Feuchtner et al. found a median waiting time for chemotherapy of 2.1 months (∼63 days) in patients diagnosed in 2012-2014. However, treatment completion rates have increased considerably since (54.1% vs 82.1%).^39^

### Self-reported barriers and access to chemotherapy

#### Acceptability

Fear of treatment and side effects was perceived as the severest constraint to accessing treatment, in line with other studies conducted in SSA.^40^^,^^41^ This may also be influenced by prior negative experiences of patients within their communities. The prevalence of unmet psychosocial and physical supportive care needs in Ethiopia is high,^42^^,^^43^ including limitations in access to adequate pain management and treatment of therapy-related side effects. Further research, particularly qualitative studies such as patient interviews or focus groups discussions, is needed to better understand the underlying drivers of treatment-related fear and to inform targeted interventions.

In this study, trust in healthcare professionals emerged as a crucial factor in chemotherapy initiation, with patients reporting more trust having increased chances of initiating chemotherapy (OR 1.76; 95% CI, 1.16-2.68). This reinforces the importance of provider-patient relationships, as the delivery of healthcare, including treatment uptake and adherence, is fundamentally based on trust.^44^^,^^45^ Poor communication, inadequate provision of patient information and negative attitude of healthcare professionals can result in dissatisfaction with or mistrust of healthcare providers.^40^^,^^41^ This, in turn, may lead to increased reliance on traditional and alternative medicine in Ethiopia^46^ and SSA^47^, potentially exacerbating hesitancy seeking conventional medical care^48^ and consecutive treatment abandonment.^49^ A recent qualitative assessment found uncertainty about the efficacy of conventional medicine among cancer patients and their relatives in Addis Ababa.^50^

Implementing structured patient communication training programs for health workers could be a crucial step in improving trust in health care providers and thus reducing barriers in accessing care.^38^ These should focus on empathetic communication, clear explanation of treatment options and side effects, and fostering patient engagement. It should be tailored to the specific communicative challenges of the Ethiopian health care system,^51^ taking into account patient needs and the pivotal role of families and communities in health decision making and psychosocial support.^51–53^

Psychosocial distress is highly prevalent in Ethiopian cancer patients,^52–54^ while psycho-oncological support remains unavailable or unutilized.^42^^,^^52^ Effective psychosocial interventions could include dedicated counselling services and integration of mental health professionals into oncology care teams. Providing culturally sensitive counselling and engaging family members in treatment discussions could further enhance treatment adherence and patient well-being.

Another promising approach is the establishment of peer support groups including cancer patients and cancer survivors. One first-of-its-kind initiative from Adama Hospital in Ethiopia demonstrates^55^ that such self-aid groups improve mental well-being, social support, community engagement, patient empowerment, and advocacy. Similar programs are being implemented in twelve hospitals, including TASH, in cooperation with our colleagues from Addis Ababa University.

#### Affordability

Patients also reported considerable problems concerning the affordability of treatment, while indirect cost of undergoing therapy (eg, loss of income, cost of childcare, etc.) were perceived as less problematic. Financial constraints and the high cost of diagnostic and treatment services are among the most commonly reported access barriers in the literature.^14^^,^^56^ Many African countries face the burden of financial toxicity of medical care, leading to a high prevalence of catastrophic health expenditure.^57^^,^^58^ Nearly all patients in the cohort relied on multiple sources of payment to cover their medical expenses, including family members and friends, suggesting a substantial financial burden.

To ease this burden, Ethiopia established a community-based health insurance system (CBHI) in 2011 focusing on rural populations, which has since expanded its coverage to over 8.7 million households.^59^ As of 2019, approximately 28.1% of the Ethiopian population were covered, with the rate in urban areas assumed to be lower (∼19%).^60^ Precise estimates of health insurance coverage in Addis Ababa are lacking, for public as for private health insurance. In our cohort, about half of patients stated having health insurance, likely reflecting higher enrolment among individuals already engaged with the healthcare system compared to the generally healthier population. Having health insurance substantially improved chemotherapy initiation, confirming that CBHI increases universal health coverage and health service utilization.^61^ Such initiatives hold promise of promoting the health-related quality of life and mitigating catastrophic health expenditure.

Interestingly, patients who perceived treatment costs as more burdensome were more likely to undergo chemotherapy. This counterintuitive finding may stem from heightened awareness of costs among those actively undergoing treatment, whereas non-recipients may not have fully grasped the financial burden.

#### Availability and accommodation

The availability of treatment services and facilities was problematic for two-thirds of participants. Common challenges such as unstable supplies and medicine stock-outs are common in SSA, and, alongside high patient volume and insufficiently trained personnel,^2^^,^^62^^,^^63^ likely contribute to treatment delays.^63^ Despite the perceived severity of waiting times as burdensome in our study, we did not find a direct correlation with the length of treatment interval.

We did not evaluate how many times patients sought care before receiving treatment. Poor coordination of care, deficiencies in patient management, as well as chemotherapy stockouts^64^ and inefficient referrals^65^ can lead to patients needing to present numerous times to health facilities before receiving treatment, which aggravates treatment delays.

Our findings underline the need for improved patient flow management, better record-keeping, and enhanced coordination among treatment facilities. Establishing electronic health records, digitalizing appointment scheduling and streamlining referral processes could reduce inefficiencies and ensure timely care.

#### Accessibility

Accessibility of transportation and the related costs are often-reported barriers that hinder access to cancer treatment in African settings.^62^^,^^66^ In this study, access to transportation was perceived as the least problematic, likely due to Addis Ababa’s expansive urbanization, wide public transport network and high density of health facilities compared to rural Ethiopia.^67^ Although the accessibility of treatment facilities was generally perceived as unproblematic, patients reporting higher levels of problems concerning access to public or private transportation had increased treatment delays.

#### Time to chemotherapy initiation

Similar to a recent study from Ethiopia,^66^ self-perceived barriers (and sociodemographic factors) did not show strong associations with the time to chemotherapy initiation. Our subgroup analysis among breast cancer patients revealed noteworthy associations. Specifically, factors such as stage, education, marital status, and whether patients underwent prior surgery impacted the time to chemotherapy treatment. This could be partly explained by time taken for postoperative recovery after breast cancer surgery.

We assumed that the combination of treatment modalities would influence the timing of chemotherapy, especially whether or not patients had surgery or consecutive radiation treatment. Hence, we carried out a stratified analysis according to treatment modalities (Table S6): patients undergoing chemotherapy (alone, neoadjuvant, or adjuvant) or chemoradiation (alone, neoadjuvant or adjuvant). In both analyses, the timing of surgery did not have a statistically significant influence on the timing of chemotherapy. Nevertheless, the results must be interpreted with caution due to the small sample sizes per group.

However, the timing of cancer operation showed an impact on chemotherapy delay when stratified by stage of disease (Table S7): patients with “Unknown stage” experienced a 2.7-fold larger delay if they underwent upfront surgery followed by chemotherapy or chemoradiation compared to those who did not. Considering that patients with missing staging are also less likely to initiate chemotherapy, these patients seem especially vulnerable and face significant (structural) barriers to accessing healthcare. It would therefore be important for future research to better understand who these patients are, what specific barriers they face, and how targeted interventions could help improve their access to care. To fully understand the factors influencing delays of treatment initiation in Ethiopia, broader research is needed.

### Strengths and limitations

To our knowledge, this is the first study to systematically assess patient-reported barriers to cancer treatment in Ethiopia. The population-based approach provides valuable insights into systemic treatment timelines and patient perspectives, contributing to evidence-based health system planning.

However, some limitations exist. Approximately one-third of the study sample was unreachable and only 58.8% of patient files were available, reducing case numbers per cancer type and affecting statistical precision. Patients lost to follow-up may have been deceased, leading to potential underrepresentation of those with poorer outcomes. The use of phone interviews, though logistically necessary, may have introduced selection bias by excluding those without mobile access and could have impacted data quality due to technical issues and distractions.

Our cohort predominantly comprised female patients (82%), reflecting the high burden of breast and cervical cancers in Addis Ababa, but limiting generalizability to male patients. Additionally, most cases were from the 2 largest tertiary hospitals, limiting applicability to lower-level facilities or areas outside the capital, where healthcare access and quality may differ.

Missing or incomplete patient records may have led to underestimation of treatment receipt and completion. Self-reported data, particularly on economic status, introduce subjectivity, while proxy interviews, may not fully capture patients’ experiences, leading to information bias. Recall bias, especially for treatment dates, remains a concern, though we mitigated this by excluding implausible dates (*n* = 28) and outliers (*n* = 9), as we assumed they were erroneously inserted data points.

## Conclusion

This is the first population-based study in Addis Ababa assessing time to chemotherapy and patient-perceived barriers across common cancers. Although chemotherapy initiation and completion rates have improved, delays from diagnosis to treatment remain. We demonstrated that trust in healthcare providers and health insurance are facilitators of access to chemotherapy. While reported barriers did not influence delays, they still act as stressors for patients and their families. Addressing these complex burdens at system level through expansion of health insurance and services and on provider–patient level may improve access to care and patient well-being.

## Supplementary Material

oyag198_Supplementary_Data

## Data Availability

The datasets generated and/or analyzed in this study are not publicly available due to ethical and privacy restrictions but can be accessed upon reasonable request to the corresponding author.
