# Supplementary material for: Access and time to chemotherapy among Ethiopian cancer patients: a population-based registry study
Source: Oncologist. 2026 May 20;31(8):oyag198. doi: 10.1093/oncolo/oyag198 (PMC13375650; doi:10.1093/oncolo/oyag198)
Supplement: oyag198_Supplementary_Data [file oyag198_supplementary_data.docx]

# Supplementary Material

**Supplementary Table 1.** **Questionnaire item for the 5 dimensions of access to care (Penchansky et al.)**

|  |  | **Question** | **Answer options** |
| --- | --- | --- | --- |
| **Availability** | The availability of a health facility within reach that is able to meet the patient’s needs and can provide the necessary treatment. | How difficult was it to find a health facility in the area that offers cancer treatment? | 1. Very problematic/ Very difficult 2. Problematic/ Somewhat difficult 3. No problem/ Not difficult |
|  |  | How difficult was it to receive treatment at the health facility due to its availability? | 1. Very problematic/ Very difficult 2. Problematic/ Somewhat difficult 3. No problem/ Not difficult |
| **Accessibility** | The patient’s chances to reach the health facility’s geographical location, including the necessary resources to do so (financial means, transportation infrastructure) | How difficult was it to access public or private transportation to reach the treatment facility? | 1. Very problematic/ Very difficult 2. Problematic/ Somewhat difficult 3. No problem/ Not difficult |
|  |  | How difficult was it to pay for the public or private transportation to reach the treatment facility? | 1. Very problematic/ Very difficult 2. Problematic/ Somewhat difficult 3. No problem/ Not difficult |
| **Accommodation** | The extent to which healthcare practitioners and their services are organized to meet the patient's needs, allowing the patient to integrate care into their everyday life | How difficult was it to receive appointments for treatment within a reasonable waiting time? | 1. Very problematic/ Very difficult 2. Problematic/ Somewhat difficult 3. No problem/ Not difficult |
|  |  | How difficult was it to make time for the treatment appointments/go on medical leave (e.g. taking time off work, childcare, leaving home, suitable office hours, other responsibilities) | 1. Very problematic/ Very difficult 2. Problematic/ Somewhat difficult 3. No problem/ Not difficult |
| **Affordability** | The patient’s financial ability to pay for the treatment and associated expenditures (e.g. loss of income due to inability to work) | How difficult was it to afford the cost of treatment (chemotherapy, drugs, hospital bills etc.)? | 1. Very problematic/ Very difficult 2. Problematic/ Somewhat difficult 3. No problem/ Not difficult |
|  |  | How difficult was it to afford the cost of being absent from home (indirect costs, loss of income, childcare etc.) | 1. Very problematic/ Very difficult 2. Problematic/ Somewhat difficult 3. No problem/ Not difficult |
| **Acceptability** | The patient's perceptions and beliefs about health care workers, treatments, facilities and the health care system in general | Were you afraid of the treatment or its side effects? | 1. Very problematic/ Very afraid 2. Somewhat problematic/ afraid 3. No problem/ Not afraid |
|  |  | Did you trust the health care workers treat your disease appropriately? | 1. Very problematic/ No trust 2. Somewhat problematic/ limited trust 3. No problem/ A lot of trust |


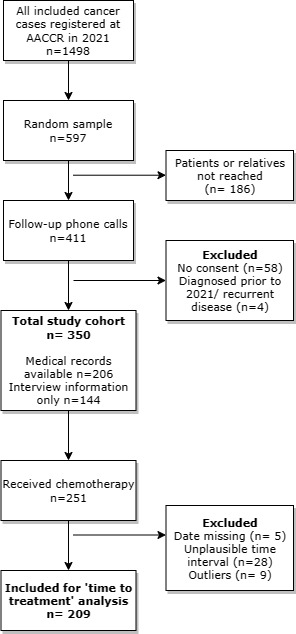


**Supplementary Figure 1. Flowchart of study participants**

**Supplementary Table 2.** **Results of logistic regression (receipt of chemotherapy) and negative binomial regression (time to chemotherapy).** OR = Odds Ratio, IRR = Incidence Rate Ratio

|  | Logistic regression (n=330) |  | Negative binomial regression (n=202) |
| --- | --- | --- | --- |

|  | n | OR (95% CI) | p |  | n | IRR (95% CI) | p |
| --- | --- | --- | --- | --- | --- | --- | --- |

| Diagnosis |  |  |  |  |  |  |  |
| --- | --- | --- | --- | --- | --- | --- | --- |

| Breast | 143 | 1 |  |  | 108 | 1 |  |
| --- | --- | --- | --- | --- | --- | --- | --- |

| Cervix | 64 | 0.07 (0.03-0.18) | <0.001 |  | 21 | 0.87 (0.46-1.65) | 0.67 |
| --- | --- | --- | --- | --- | --- | --- | --- |

| Colorectum | 60 | 0.38 (0.12-1.19) | 0.10 |  | 39 | 1.41 (0.82-2.39) | 0.21 |
| --- | --- | --- | --- | --- | --- | --- | --- |

| Gastric | 26 | 0.21 (0.05-0.86) | 0.03 |  | 15 | 1.02 (0.48-2.18) | 0.95 |
| --- | --- | --- | --- | --- | --- | --- | --- |

| Oesophagus | 17 | 0.10 (0.02-0.53) | 0.01 |  | 5 | 0.61 (0.19-2.01) | 0.42 |
| --- | --- | --- | --- | --- | --- | --- | --- |

| Ovary | 20 | 1.00 (0.19-5.33) | 1.00 |  | 14 | 1.11 (0.51-2.44) | 0.79 |
| --- | --- | --- | --- | --- | --- | --- | --- |
| Age | 330 | 1.01 (0.99-1.04) | 0.38 |  |  | 1.00 (0.99-1.02) | 0.80 |
| Sex |  |  |  |  |  |  |  |
| Male | 57 | 1 |  |  | 31 | 1 |  |
| Female | 273 | 0.97 (0.37-2.55) | 0.95 |  | 171 | 0.96 (0.55-1.68) | 0.89 |
| Stage |  |  |  |  |  |  |  |
| Early stage (I+II) | 59 | 1 | 0.03 |  | 40 | 1 |  |
| Stage III | 95 | 0.41 (0.13-1.31) | 0.13 |  | 65 | 1.13 (0.71-1.78) | 0.61 |
| Stage IV | 77 | 0.41 (0.13-1.34) | 0.14 |  | 41 | 1.24 (0.71-2.14) | 0.45 |
| Unknown | 99 | 0.19 (0.06-0.59) | <0.001 |  | 56 | 1.10 (0.68-1.77) | 0.70 |
| Marital status |  |  |  |  |  |  |  |
| Married | 237 | 1 |  |  | 148 | 1 |  |
| Unmarried | 93 | 1.49 (0.70-3.19) | 0.30 |  | 54 | 0.86 (0.59-1.25) | 0.42 |
| Education level |  |  |  |  |  |  |  |
| No schooling | 57 | 1 | 0.66 |  | 33 | 1 |  |
| Primary | 111 | 0.67 (0.26-1.71) | 0.40 |  | 61 | 1.14 (0.68-1.90) | 0.62 |
| Secondary | 103 | 0.89 (0.31-2.56) | 0.83 |  | 69 | 1.06 (0.63-1.78) | 0.83 |
| Tertiary | 59 | 1.19 (0.36-3.98) | 0.78 |  | 39 | 1.00 (0.54-1.85) | 0.99 |
| Self-perceived wealth |  |  |  |  |  |  |  |
| Poor | 130 | 1 |  |  | 77 | 1 |  |
| Middle class | 200 | 1.23 (0.57-2.65) | 0.61 |  | 125 | 0.86 (0.59-1.24) | 0.41 |
| Health insurance |  |  |  |  |  |  |  |
| No | 155 | 1 |  |  | 84 | 1 |  |
| Yes | 175 | 2.70 (1.33-5.50) | 0.01 |  | 118 | 0.86 (0.61-1.23) | 0.42 |
| Surgery prior to chemotherapy | | |  |  |  |  |  |
| No |  |  |  |  | 67 | 1 |  |
| Yes |  |  |  |  | 135 | 1.27 (0.87-1.86) | 0.22 |
| Availability |  |  |  |  |  |  |  |
| Treatment facilities | 330 | 1.47 (0.83-2.62) | 0.19 |  | 202 | 1.03 (0.75-1.43) | 0.84 |
| Treatment services | 330 | 1.73 (0.96-3.10) | 0.07 |  | 202 | 0.95 (0.74-1.23) | 0.72 |
| Accessibility |  |  |  |  |  |  |  |
| Transportation | 330 | 0.80 (0.36-1.77) | 0.58 |  | 202 | 0.74 (0.53-1.03) | 0.07 |
| Cost of transport | 330 | 0.91 (0.49-1.68) | 0.76 |  | 202 | 1.03 (0.80-1.34) | 0.81 |
| Accommodation |  |  |  |  |  |  |  |
| Wait times | 330 | 1.07 (0.59-1.95) | 0.82 |  | 202 | 0.92 (0.67-1.28) | 0.63 |
| Ability to leave home | 330 | 1.06 (0.56-2.01) | 0.85 |  | 202 | 1.05 (0.73-1.51) | 0.80 |
| Affordability |  |  |  |  |  |  |  |
| Treatment cost | 330 | 0.20 (0.11-0.39) | <0.001 |  | 202 | 0.95 (0.69-1.31) | 0.76 |
| Indirect cost | 330 | 1.03 (0.49-2.16) | 0.93 |  | 202 | 0.98 (0.69-1.40) | 0.93 |
| Acceptability |  |  |  |  |  |  |  |
| Fear of treatment | 330 | 1.27 (0.72-2.27) | 0.41 |  | 202 | 0.96 (0.71-1.28) | 0.76 |
| Trust in health care | 330 | 1.76 (1.16-2.68) | 0.01 |  | 202 | 1.10 (0.87-1.39) | 0.44 |

**Supplementary Table 3. Results of the Cox regression and Fine and Gray regression modelling the hazard of receiving chemotherapy.**

|  | Cox regression (n=315) | | |  | Fine and Gray regression (n=315) | | |
| --- | --- | --- | --- | --- | --- | --- | --- |
|  | **n** | **HR (95% CI)** | **p** |  | **n** | **SHR (95%CI)** | **p** |
| Diagnosis |  |  |  |  |  |  |  |
| Breat (Ref.) | 132 | 1.00 |  |  | 132 | 1.00 |  |
| Cervix | 64 | 0.30 (0.19-0.50) | <0.001 |  | 64 | 0.30 (0.18-0.49) | <0.001 |
| Colorectum | 58 | 0.79 (0.49-1.29) | 0.35 |  | 58 | 0.73 (0.46-1.18) | 0.20 |
| Stomach | 25 | 0.65 (0.33-1.28) | 0.21 |  | 25 | 0.54 (0.27-1.08) | 0.08 |
| Oesophagus | 17 | 0.38 (0.16-0.87) | 0.02 |  | 17 | 0.38 (0.15-0.94) | 0.04 |
| Ovary | 19 | 0.63 (0.32-1.27) | 0.20 |  | 19 | 0.68 (0.32-1.41) | 0.30 |
| Age | 315 | 1.00 (0.99-1.01) | 0.87 |  | 315 | 1.00 (0.99-1.01) | 0.86 |
| Sex |  |  |  |  |  |  |  |
| Female (Ref.) | 261 | 1.00 |  |  | 261 | 1.00 |  |
| Male | 54 | 0.93 (0.58-1.49) | 0.78 |  | 54 | 0.94 (0.58-1.53) | 0.81 |
| Stage |  |  |  |  |  |  |  |
| Stage I+II (Ref.) | 51 | 1.00 |  |  | 51 | 1.00 |  |
| Stage III | 92 | 0.85 (0.57-1.28) | 0.45 |  | 92 | 0.84 (0.56-1.24) | 0.38 |
| Stage IV | 73 | 0.95 (0.59-1.54) | 0.84 |  | 73 | 0.85 (0.55-1.33) | 0.49 |
| Stage Unknown | 99 | 0.63 (0.41-0.96) | 0.03 |  | 99 | 0.60 (0.40-0.89) | 0.01 |
| Marital status |  |  |  |  |  |  |  |
| Married (Ref.) | 231 | 1.00 |  |  | 231 | 1.00 |  |
| Unmarried | 84 | 1.20 (0.84-1.70) | 0.32 |  | 84 | 1.22 (0.85-1.76) | 0.28 |
| Education level |  |  |  |  |  |  |  |
| No schooling (Ref.) | 55 | 1.00 |  |  | 55 | 1.00 |  |
| Primary education | 107 | 0.84 (0.54-1.31) | 0.44 |  | 107 | 0.83 (0.53-1.32) | 0.44 |
| Secondary education | 96 | 0.98 (0.62-1.57) | 0.95 |  | 96 | 0.99 (0.62-1.56) | 0.96 |
| Tertiary education | 57 | 1.12 (0.65-1.93) | 0.69 |  | 57 | 1.20 (0.70-2.06) | 0.50 |
| Self-perceived wealth | |  |  |  |  |  |  |
| Poor (Ref.) | 128 | 1.00 |  |  | 128 | 1.00 |  |
| Middle class | 187 | 1.34 (0.94-1.92) | 0.11 |  | 187 | 1.34 (0.97-1.84) | 0.07 |
| Health insurance |  |  |  |  |  |  |  |
| No Insurance (Ref.) | 145 | 1.00 |  |  | 145 | 1.00 |  |
| Health Insurance | 170 | 1.50 (1.09-2.06) | 0.01 |  | 170 | 1.58 (1.19-2.10) | 0.002 |
| Availability |  |  |  |  |  |  |  |
| Treatment facilities | 315 | 1.00 (0.78-1.29) | 0.98 |  | 315 | 1.04 (0.83-1.31) | 0.71 |
| Treatment services | 315 | 1.15 (0.89-1.49) | 0.29 |  | 315 | 1.13 (0.88-1.45) | 0.34 |
| Accessibility |  |  |  |  |  |  |  |
| Transportation | 315 | 1.08 (0.78-1.50) | 0.65 |  | 315 | 1.13 (0.82-1.55) | 0.45 |
| Cost of transport | 315 | 0.91 (0.71-1.17) | 0.47 |  | 315 | 0.91 (0.71-1.16) | 0.43 |
| Accommodation |  |  |  |  |  |  |  |
| Waittimes | 315 | 1.08 (0.80-1.44) | 0.62 |  | 315 | 1.13 (0.83-1.54) | 0.44 |
| Medical leave | 315 | 1.09 (0.79-1.50) | 0.60 |  | 315 | 1.13 (0.81-1.56) | 0.48 |
| Affordability |  |  |  |  |  |  |  |
| Treatment cost | 315 | 0.60 (0.45-0.79) | 0.00 |  | 315 | 0.58 (0.44-0.76) | <0.001 |
| Indirect cost | 315 | 0.93 (0.67-1.30) | 0.67 |  | 315 | 0.89 (0.65-1.21) | 0.45 |
| Acceptability |  |  |  |  |  |  |  |
| Fear of treatment | 315 | 0.99 (0.77-1.27) | 0.92 |  | 315 | 1.01 (0.80-1.28) | 0.91 |
| Trust in health care | 315 | 1.23 (1.00-1.51) | 0.05 |  | 315 | 1.24 (1.01-1.52) | 0.04 |

HR = Hazard Ratio, SHR= Subhazard Ratio

**Supplementary Table 4. Subgroup analysis including breast cancer patients only. Results of logistic regression (receipt of chemotherapy) and negative binomial regression (time to chemotherapy)**

|  | | Logistic regression (n=143) | | | Negative binomial regression (n=108) | | |
| --- | --- | --- | --- | --- | --- | --- | --- |
|  | | **OR (95% CI)** | **p** | | **IRR (95% CI)** | | **p** |
| Age | | 0.97 (0.89-1.06) | 0.47 | | 1.01 (0.99-1.03) | | 0.62 |
| Sex | |  |  | |  | |  |
| Male | | 1.00 | . | | 1.00 | | . |
| Female | | 3.73 (0.17-81.77) | 0.40 | | 2.17 (0.76-6.24) | | 0.15 |
| Stage | |  |  | |  | |  |
| Early stage (I + II) | | 1.00 | 0.15 | | 1.00 | | . |
| Stage III | | 0.04 (0.00-1.45) | 0.08 | | 1.61 (0.88-2.93) | | 0.12 |
| Stage IV | | 0.56 (0.01-43.97) | 0.79 | | 2.60 (0.96-7.01) | | 0.06 |
| Unknown | | 0.03 (0.00-0.97) | 0.05 | | 1.45 (0.75-2.80) | | 0.27 |
| Education | |  |  | |  | |  |
| No schooling | | 1.00 | 0.91 | | 1.00 | | . |
| Primary | | 3.31 (0.10-106.13) | 0.50 | | 0.46 (0.20-1.05) | | 0.06 |
| Secondary | | 3.16 (0.13-77.97) | 0.48 | | 0.61 (0.28-1.35) | | 0.22 |
| Tertiary | | 3.55 (0.08-160.99) | 0.52 | | 0.56 (0.23-1.39) | | 0.21 |
| Self-perceived wealth | |  |  | |  | |  |
| Poor | | 1.00 | . | | 1.00 | | . |
| Middle class | | 0.73 (0.05-11.83) | 0.83 | | 0.94 (0.53-1.65) | | 0.82 |
| Marital status | |  |  | |  | |  |
| Married | |  |  | | 1.00 | | . |
| Unmarried | | 1.98 (0.08-49.63) | 0.68 | | 0.52 (0.28-0.97) | | 0.04 |
| Health insurance | |  |  | |  | |  |
| No | | 1.00 | . | | 1.00 | | . |
| Yes | | 1.41 (0.13-15.87) | 0.78 | | 0.90 (0.55-1.46) | | 0.67 |
| Surgery before chemotherapy |  | | |  | |  | |
| No | |  |  | | 1.00 | | . |
| Surgery prior | |  |  | | 1.75 (0.97-3.13) | | 0.06 |
| Availability | |  |  | |  | |  |
| Treatment facilities | | 0.42 (0.07-2.67) | 0.36 | | 1.01 (0.66-1.54) | | 0.98 |
| Treatment services | | 0.47 (0.06-3.59) | 0.47 | | 0.93 (0.62-1.37) | | 0.70 |
| Accessibility | |  |  | |  | |  |
| Transportation | | 0.00 (0.00-.) | 1.00 | | 0.66 (0.38-1.17) | | 0.15 |
| Cost of transport | | 1.15 (0.18-7.17) | 0.88 | | 1.22 (0.87-1.69) | | 0.25 |
| Accommodation | |  |  | |  | |  |
| Wait times | | 1.51 (0.21-10.87) | 0.68 | | 0.86 (0.54-1.38) | | 0.54 |
| Medical leave | | 1.07 (0.14-8.25) | 0.95 | | 1.18 (0.72-1.94) | | 0.50 |
| Affordability | |  |  | |  | |  |
| Treatment cost | | 0.05 (0.01-0.41) | 0.01 | | 1.04 (0.65-1.66) | | 0.86 |
| Indirect cost | | 2.09 (0.16-27.31) | 0.57 | | 1.08 (0.67-1.75) | | 0.75 |
| Acceptability | |  |  | |  | |  |
| Fear of treatment | | 0.34 (0.06-1.87) | 0.21 | | 0.93 (0.60-1.42) | | 0.72 |
| Trust in health care | | 9.59 (1.96-46.96) | 0.01 | | 1.29 (0.92-1.82) | | 0.14 |

OR = Odds Ratio, IRR = Incidence Rate Ratio

**Supplementary Table 5. Results of Logistic Regression assessing the relationship between responding person (Interviewee being patient vs next-of-kin) and the scores in the 10 self-reported barriers to care.**

| Predictor | OR | 95% CI (Lower) | 95% CI (Upper) |
| --- | --- | --- | --- |
| Constant | 1.520 | 0.683 | 3.387 |
| Availability of treatment facilities | 1.117 | 0.750 | 1.664 |
| Availability of treatment services | 0.802 | 0.550 | 1.170 |
| Accessibility of transportation | 0.744 | 0.441 | 1.257 |
| Affordability of transport cost | 1.243 | 0.852 | 1.814 |
| Accommodation of wait times | 0.772 | 0.510 | 1.168 |
| Ability to make time for appointment | 0.967 | 0.599 | 1.559 |
| Affordability of treatment cost | 1.411 | 0.936 | 2.127 |
| Affordability of indirect cost | 1.332 | 0.835 | 2.125 |
| Fear of treatment | 0.819 | 0.564 | 1.189 |
| Trust in health care | 0.794 | 0.579 | 1.088 |

**Supplementary Table 6.** **Negative binomial regression (time to chemotherapy initiation) stratified according to treatment modality (chemotherapy vs. chemoradiation)**. IRR = Incidence Rate Ratios

|  | Chemotherapy (chemotherapy alone, neo/adjuvant chemotherapy; n=156) | | | Chemoradiation (chemoradiation alone, neo/adjuvant chemoradiation; n=46) | | |
| --- | --- | --- | --- | --- | --- | --- |
|  | **n** | **IRR (Lower CI-Upper CI)** | **p** | **n** | **IRR (Lower CI-Upper CI)** | **p** |
| Diagnosis |  |  |  |  |  |  |
| Breast | 81 | 1.0 |  | 27 | 1.0 |  |
| Cervix | 7 | 1.7 (0.65-4.43) | 0.28 | 14 | 0.34 (0.08-1.50) | 0.16 |
| Colorectum | 34 | 1.57 (0.88-2.81) | 0.13 | 5 | 1.16 (0.17-7.78) | 0.88 |
| Gastric | 15 | 0.96 (0.41-2.28) | 0.94 | - | - | - |
| Oesophagus | 5 | 0.42 (0.12-1.48) | 0.18 | - | - | - |
| Ovary | 14 | 0.87 (0.37-2.00) | 0.74 | - | - | - |
| Age | 156 | 1.0 (0.99-1.02) | 0.95 | 46 | 1.02 (0.96-1.08) | 0.47 |
| Sex |  |  |  |  |  |  |
| Male | 28 | 1.0 |  | 3 | 1.0 |  |
| Female | 128 | 0.73 (0.39-1.34) | 0.31 | 43 | 1.54 (0.13-18.56) | 0.74 |
| Stage |  |  |  |  |  |  |
| Early stage | 32 | 1.0 |  | 8 | 1.0 |  |
| Stage III | 42 | 0.96 (0.56-1.65) | 0.88 | 23 | 0.81 (0.19-3.54) | 0.78 |
| Stage IV | 32 | 0.95 (0.49-1.82) | 0.88 | 9 | 0.91 (0.15-5.44) | 0.92 |
| Unknown stage | 50 | 1.1 (0.64-1.88) | 0.74 | 6 | 1.02 (0.22-4.78) | 0.98 |
| Marital status | | |  |  |  |  |
| Married | 113 | 1.0 |  | 35 | 1.0 |  |
| Unmarried | 43 | 1.04 (0.68-1.60) | 0.86 | 11 | 0.58 (0.16-2.10) | 0.41 |
| Education level | | | | | | |
| No schooling | 28 | 1.0 |  | 5 | 1.0 |  |
| Primary | 45 | 0.83 (0.47-1.49) | 0.54 | 16 | 2.15 (0.33-13.93) | 0.42 |
| Secondary | 55 | 1.02 (0.57-1.80) | 0.95 | 14 | 1.52 (0.23-10.01) | 0.66 |
| Tertiary | 28 | 0.88 (0.44-1.76) | 0.71 | 11 | 1.81 (0.21-15.69) | 0.59 |
| Wealth |  |  |  |  |  |  |
| Poor | 64 | 1.0 |  | 13 | 1.0 |  |
| Middle income | 92 | 0.8 (0.52-1.22) | 0.3 | 33 | 0.42 (0.07-2.66) | 0.36 |
| Insurance status | | | | | | |
| No insurance | 61 | 1.0 |  | 23 | 1.0 |  |
| Insurance | 95 | 0.68 (0.44-1.03) | 0.07 | 23 | 0.89 (0.27-2.92) | 0.85 |
| Surgical treatment | | | | | | |
| No prior surgery | 44 | 1.0 |  | 23 | 1.0 |  |
| Prior surgery | 112 | 1.27 (0.82-1.95) | 0.28 | 23 | 1.28 (0.37-4.39) | 0.69 |
| Better Access regarding | | | | | | |
| Availability | | | | | | |
| Treatment facilities | 156 | 1.06 (0.73-1.55) | 0.75 | 46 | 0.84 (0.32-2.21) | 0.73 |
| Treatment services | 156 | 1.02 (0.76-1.39) | 0.87 | 46 | 0.66 (0.24-1.79) | 0.41 |
| Accessibility | | | | | | |
| Transportation | 156 | 0.72 (0.49-1.06) | 0.09 | 46 | 1.28 (0.48-3.38) | 0.62 |
| Cost of transport | 156 | 1.03 (0.78-1.36) | 0.84 | 46 | 0.89 (0.43-1.81) | 0.74 |
| Accommodation | | | | | | |
| Wait time | 156 | 0.94 (0.65-1.38) | 0.77 | 46 | 1.18 (0.47-2.98) | 0.72 |
| Ability to leave home | 156 | 1.08 (0.69-1.71) | 0.73 | 46 | 1.27 (0.47-3.40) | 0.64 |
| Affordability | | | | | | |
| Treatment cost | 156 | 0.98 (0.67-1.44) | 0.92 | 46 | 0.99 (0.44-2.21) | 0.98 |
| Indirect cost | 156 | 0.84 (0.54-1.29) | 0.42 | 46 | 1.49 (0.62-3.59) | 0.37 |
| Acceptability | | | | | | |
| Fear | 156 | 1.15 (0.81-1.64) | 0.43 | 46 | 0.66 (0.24-1.83) | 0.42 |
| Trust in health care | 156 | 0.99 (0.75-1.31) | 0.95 | 46 | 1.33 (0.59-2.99) | 0.49 |

**Supplementary Table 7.** **Negative binomial regression (time to chemotherapy initiation) stratified according to stage of disease at diagnosis**. IRR = Incidence Rate Ratios

|  | Early stage (I + II) (n=40) | | | Stage III (n=65) | | | Stage IV (n=41) | | | Unknown stage (n=56) | | |
| --- | --- | --- | --- | --- | --- | --- | --- | --- | --- | --- | --- | --- |
|  | **n** | **IRR (95%CI)** | **p** | **n** | **IRR (95%CI)** | **p** | **n** | **IRR (95%CI)** | **p** | **n** | **IRR (Lower CI-Upper CI)** | **p** |
| Diagnosis |  |  |  |  |  |  |  |  |  |  |  |  |
| Breast | 24 | 1.0 |  | 43 | 1.0 |  | 9 | 1.0 |  | 32 | 1.0 |  |
| Cervix | 7 | 0.51 (0.46-4.64) | 0.51 | 6 | 0.24 (0.05-1.22) | 0.08 | 5 | 0.95 (0.18-5.11) | 0.95 | 3 | 1.95 (0.34-11.01) | 0.45 |
| Colorectum | 6 | 0.31 (0.05-1.89) | 0.21 | 14 | 1.46 (0.47-4.53) | 0.51 | 10 | 0.61 (0.11-3.47) | 0.57 | 9 | 4.11 (1.14-14.8) | 0.03 |
| Gastric | - | - | - | 1 | 0.86 (0.04-17.36) | 0.92 | 11 | 0.49 (0.08-3.13) | 0.45 | 3 | 1.09 (0.11-10.58) | 0.94 |
| Oesophagus | - | - | - | - | - | 0.26 | 4 | 0.13 (0.01-3.2) | 0.21 | 1 | 0.59 (0.04-7.78) | 0.69 |
| Ovary | 3 | 4.17 (0.3-57.91) | 0.29 | 1 | 0.24 (0.02-2.88) | - | 2 | 0.87 (0.04-20.11) | 0.93 | 8 | 0.58 (0.13-2.63) | 0.48 |
| Age | 40 | 0.99 (0.94-1.04) | 0.58 | 65 | 1.02 (0.98-1.05) | 0.38 | 41 | 0.98 (0.93-1.03) | 0.39 | 56 | 1.0 (0.97-1.02) | 0.86 |
| Sex |  |  |  |  |  |  |  |  |  |  |  |  |
| Male | 2 | 1.0 |  | 8 | 1.0 |  | 11 | 1.0 |  | 10 | 1.0 |  |
| Female | 38 | 0.14 (0.01-1.68) | 0.12 | 57 | 2.11 (0.51-8.65) | 0.3 | 30 | 0.74 (0.16-3.54) | 0.71 | 46 | 1.01 (0.36-2.83) | 0.99 |
| Marital status |  |  |  |  |  |  |  |  |  |  |  |  |
| Married | 29 | 1.0 |  | 49 | 1.0 |  | 29 | 1.0 |  | 41 | 1.0 |  |
| Unmarried | 11 | 0.46 (0.12-1.71) | 0.25 | 16 | 0.94 (0.39-2.27) | 0.9 | 12 | 0.66 (0.15-2.89) | 0.58 | 15 | 0.46 (0.15-1.39) | 0.17 |
| Education level |  |  |  |  |  |  |  |  |  |  |  |  |
| No schooling | 8 | 1.0 |  | 9 | 1.0 |  | 9 | 1.0 |  | 7 | 1.0 |  |
| Primary | 10 | 1.68 (0.48-5.94) | 0.42 | 15 | 1.34 (0.34-5.24) | 0.67 | 14 | 1.15 (0.19-6.91) | 0.88 | 22 | 0.66 (0.16-2.66) | 0.56 |
| Secondary | 15 | 0.97 (0.3-3.08) | 0.96 | 24 | 1.83 (0.42-8.01) | 0.42 | 11 | 1.16 (0.12-11.22) | 0.90 | 19 | 0.72 (0.19-2.7) | 0.62 |
| Tertiary | 7 | 0.36 (0.05-2.48) | 0.30 | 27 | 1.93 (0.43-8.74) | 0.39 | 7 | 2.8 (0.41-19.08) | 0.29 | 8 | 0.34 (0.06-1.86) | 0.21 |
| Wealth |  |  |  |  |  |  |  |  |  |  |  |  |
| Poor | 12 | 1.0 |  | 28 | 1.0 |  | 16 | 1.0 |  | 21 | 1.0 |  |
| Middle class /rich | 28 | 1.13 (0.32-3.97) | 0.85 | 37 | 0.66 (0.25-1.74) | 0.4 | 25 | 0.95 (0.29-3.07) | 0.93 | 35 | 1.43 (0.54-3.77) | 0.47 |
| Insurance status |  |  |  |  |  |  |  |  |  |  |  |  |
| Uninsured | 16 | 1.0 |  | 20 | 1.0 |  | 20 | 1.0 |  | 28 | 1.0 |  |
| Insured | 24 | 0.69 (0.17-2.78) | 0.60 | 45 | 1.12 (0.53-2.35) | 0.77 | 21 | 0.43 (0.14-1.28) | 0.13 | 28 | 0.94 (0.41-2.17) | 0.89 |
| Surgical treatment |  |  |  |  |  |  |  |  |  |  |  |  |
| No prior surgery | 10 | 1.0 |  | 17 | 1.0 |  | 26 | 1.0 |  | 14 | 1.0 |  |
| Prior surgery | 30 | 0.65 (0.11-3.84) | 0.63 | 48 | 0.89 (0.34-2.3) | 0.81 | 15 | 1.99 (0.57-6.88) | 0.28 | 42 | 2.74 (1.14-6.62) | 0.02 |
| Better Access Regarding | | | | | | | | | | | | |
| Availability |  |  |  |  |  |  |  |  |  |  |  |  |
| Treatment facilities | 40 | 0.42 (0.14-1.29) | 0.13 | 65 | 1.28 (0.65-2.52) | 0.48 | 41 | 0.53 (0.19-1.49) | 0.23 | 56 | 1.26 (0.62-2.55) | 0.53 |
| Treatment services | 40 | 1.72 (0.88-3.36) | 0.12 | 65 | 0.66 (0.36-1.22) | 0.18 | 41 | 0.91 (0.34-2.44) | 0.85 | 56 | 1.05 (0.56-1.97) | 0.87 |
| Accessibility |  |  |  |  |  |  |  |  |  |  |  |  |
| Transportation | 40 | 1.51 (0.5-4.58) | 0.46 | 65 | 0.73 (0.33-1.65) | 0.45 | 41 | 0.62 (0.09-4.23) | 0.63 | 56 | 0.74 (0.22-2.45) | 0.62 |
| Cost of transport | 40 | 0.85 (0.42-1.75) | 0.67 | 65 | 1.4 (0.86-2.28) | 0.18 | 41 | 1.29 (0.15-11.38) | 0.82 | 56 | 0.84 (0.47-1.51) | 0.55 |
| Accommodation |  |  |  |  |  |  |  |  |  |  |  |  |
| Wait time | 40 | 0.66 (0.23-1.86) | 0.43 | 65 | 0.9 (0.45-1.79) | 0.76 | 41 | 1.12 (0.43-2.92) | 0.81 | 56 | 1.05 (0.49-2.26) | 0.89 |
| Ability to leave home | 40 | 1.29 (0.4-4.17) | 0.67 | 65 | 0.69 (0.33-1.48) | 0.34 | 41 | 2.02 (0.58-6.97) | 0.27 | 56 | 0.95 (0.41-2.21) | 0.91 |
| Affordability |  |  |  |  |  |  |  |  |  |  |  |  |
| Treatment cost | 40 | 1.43 (0.41-4.92) | 0.57 | 65 | 0.56 (0.25-1.26) | 0.16 | 41 | 1.14 (0.46-2.82) | 0.78 | 56 | 0.6 (0.27-1.32) | 0.21 |
| Indirect cost | 40 | 0.84 (0.23-3.05) | 0.80 | 65 | 2.55 (1.36-4.79) | 0.0 | 41 | 0.8 (0.27-2.39) | 0.69 | 56 | 0.48 (0.21-1.08) | 0.08 |
| Acceptability |  |  |  |  |  |  |  |  |  |  |  |  |
| Fear | 40 | 1.4 (0.54-3.62) | 0.49 | 65 | 0.95 (0.52-1.73) | 0.87 | 41 | 1.92 (0.53-6.89) | 0.32 | 56 | 0.9 (0.46-1.78) | 0.77 |
| Trust in health care | 40 | 1.17 (0.43-3.2) | 0.76 | 65 | 0.84 (0.49-1.44) | 0.54 | 41 | 1.4 (0.75-2.59) | 0.29 | 56 | 1.46 (0.83-2.58) | 0.19 |
